# Absence of the Epithelial Glycocalyx As Potential Tumor Marker for the Early Detection of Colorectal Cancer

**DOI:** 10.1371/journal.pone.0168801

**Published:** 2016-12-29

**Authors:** Katrin Ramaker, Steffen Bade, Niels Röckendorf, Barbara Meckelein, Ekkehard Vollmer, Holger Schultz, Günter-Willi Fröschle, Andreas Frey

**Affiliations:** 1 Division of Mucosal Immunology and Diagnostics, Priority Area Asthma & Allergy, Research Center Borstel, Airway Research Center North (ARCN), Member of the German Center for Lung Research (DZL), Borstel, Germany; 2 Division of Clinical and Experimental Pathology, Department of Medicine, Research Center Borstel, Airway Research Center North (ARCN), Member of the German Center for Lung Research (DZL), Borstel, Germany; 3 Asklepios Clinic, Bad Oldesloe, Germany; University of Campinas, BRAZIL

## Abstract

Detection of cancer at an early stage is pivotal for successful treatment and long term survival, yet early diagnosis requires sensitive and specific markers that can be easily detected by screening procedures. Differences in the surface structure of tumor and healthy cells, if sufficiently pronounced and discernible, may serve that purpose. We analyzed the luminal surface of healthy and neoplastic human colorectal tissues for the presence and architecture of the glycocalyx—a dense network of highly glycosylated proteins—using transmission electron microscopy. The ultrastructural analyses showed that 93% of healthy mucosae were covered by an intact glycocalyx. Contrarily, on over 90% of the surface of neoplastic cells the glycocalyx was absent. The sensitivity and specificity of our marker “absence of a glycocalyx” are excellent, being 91% (83–96%) and 96% (89–99%) for adenocarcinomas and 94% (73–100%) and 92% (85–97%) for precancerous polyps (means and 95% confidence intervals). Using a cell culture model we could demonstrate that a particulate probe targeting a cell surface receptor usually concealed beneath the glycocalyx can bind selectively to glycocalyx-free areas of a tumor cell layer. We propose that the absence of a glycocalyx may serve as novel type of tumor marker. If the absence of the glycocalyx can be detected e.g. via binding of imaging probes to non-shielded surface receptors of anomalously differentiated cells, this tumor marker could be used to enable early diagnosis of colorectal cancer.

## Introduction

Changes in gene expression which in turn trigger an anomalous differentiation state are a hallmark of cancer [1]. Consequently, scientists are searching for such anomalies to be used for detection, diagnosis and prognosis of cancer. Classical tumor markers are endogenous molecules that are produced in increased levels by tumor cells themselves or by healthy cells in response to cancerous conditions and that can be identified in body fluids, such as plasma or urine, or in tissues. Although the ease of collection of most body fluids renders them prime targets for screening purposes, a major drawback of most classical tumor markers is that they are not specific for a particular type of cancer. Furthermore they are not suitable to locate the actual site of malignancy. Exact localization of tumors could be achieved using imaging techniques, however, this approach requires sensitive and specific imaging probes that discriminate between healthy and malignant tissue. Hence, a major challenge for early detection of tumors via imaging is the identification of suitable surface markers that can be targeted by appropriate imaging probes to allow the localization of cancerous lesions.

In search for a potential tumor marker which is suitable for detection by imaging technologies and allows the localization of clustered cancerous cells within the body and their discrimination from healthy cells we focused on morphological differences between cancerous cells and their healthy neighbors. When we analyzed Caco-2_BBe2_ cells, a colon carcinoma-derived *in vitro* model system for human enterocytes, by ultrastructural examination we found that they lack a mature glycocalyx on their apical cell membrane [2]. This is in strong contrast to healthy intestinal enterocytes where the apical surface is covered by a dense network of highly glycosylated proteins and lipids anchored in the epithelial cell membrane [3, 4]. As the Caco-2 cells resemble poorly differentiated enterocytes, we reckoned that, if this feature was verifiable *in vivo*, it might be utilized for the diagnosis of colon carcinoma. That would require that it be possible to specifically detect the selective absence of a glycocalyx on cancerous intestinal cells. To address this diagnostic challenge, we developed a strategy based on the hypothesis that ubiquitous epithelial surface receptors which are usually shielded by the glycocalyx on healthy cells become more accessible on the non-, de- or ill-differentiated intestinal tumor cells. We conceptualized that such receptors may then be targeted specifically by particulate probes that would not be able to penetrate the intact glycocalyx of a healthy gut epithelium. This way, the glycocalyx would represent a nonclassical type of tumor marker, because the absence of this feature under malignant conditions of the cells is utilized for detection of the neoplasia.

Kaye et al. reported that in adenomatous colonic mucosa the epithelium consists of undifferentiated cells with few small, irregularly spaced or completely absent microvilli [5], but as yet there are no investigations regarding the presence or absence of an apical glycocalyx on neoplastic human colorectal tissue. Thus the aim of this study was to check whether an intact glycocalyx is missing on the apical surface of anomalously differentiated intestinal epithelial cells *in vivo* and if such a feature could serve as diagnostic imaging marker for colorectal cancer (CRC) and its precursory stages. To this purpose, tissues isolated from adenocarcinomas, adenomatous polyps and healthy colorectal mucosae were analyzed for the presence and architecture of the luminal glycocalyx on neoplastic and healthy epithelium. Furthermore, a model system was established to evaluate the potential of glycocalyx differences to be used as a diagnostic marker by targeting a cell membrane receptor with particulate probes.

## Materials and Methods

### Patients

Fifteen patients diagnosed with CRC (7 women, 8 men, aged 50 to 88 years) were included in this study (Table 1). The study was approved by the ethics committee of the University of Lübeck and patients provided written informed consent.

**Table 1:** Clinical details of patients and analysis of glycocalyx layer.

Patient No.	Age	Sex	Location of the AdCa	Isolated tissue			Mean height of existent glycocalyx in healthy tissue^a^ [nm]	Coverage of tissue with intact glycocalyx [%]		
				Healthy	AdCa	Adenoma		Healthy	**AdCa**	**Adenoma**
1	80	M	SC	X	X		1,530	100	0	
2	72	F	cecum	X	X	X	666	72.5	0	33.4
3	76	M	SC	X	X	X	1,544	100	0	0
4	80	F	cecum		X				0	
5	88	F	cecum & TC	X / X	X / X		746 / 968	99.6 / 100	0 / 0	
6	71	F	SC	X	X		1,230	99.2	24.2	
7	73	M	LF	X	X		1,364	100	50.8	
8	85	M	SC	X	X		1,130	100	10.4	
9	50	M	DC	X	X	X	1,005	100	22.8	9.7
10	75	F	SC	X	X		1,454	100	0	
11	87	F	SC	X	X		1,113	98.3	0	
12	72	M	rectum	X	X		1,073	100	0	
13	85	F	AC	X	X		1,232	86.6	0	
14	81	M	LF	X	X		1,095	90.8	0	
15	62	M	TC	X	X		963	59.3	51.0	

Abbreviations: AdCa = adenocarcinoma; M = male; F = female; SC = sigmoid colon; TC = transverse colon; LF = left flexure; DC = descending colon; AC = ascending colon.

^a^Mean of 20–30 measurements

All patients underwent intestinal surgery at the Asklepios Clinic Bad Oldesloe (Germany). Resected intestinal tissues, removed due to the respective diagnosed disease, were immediately placed on ice and inspected by a pathologist. Based on visual examination of the resected tissues, the mucosal tissues were classified into one of three categories: healthy mucosa, adenoma or adenocarcinoma. For each patient samples from all tissue categories available were taken and prepared for ultrastructural examination.

### Light and electron microscopy of tissues

The tissue samples were prepared for glycocalyx visualization by transmission electron microscopy as described previously [6]. Briefly, the specimens were cut into small blocks (approximately 2 mm x 2 mm x 4 mm) and fixed in a 1:1 mixture of 1% (w/v) osmium tetroxide containing 7.5 mg/ml of potassium ferrocyanide and a 5% (v/v) solution of glutaraldehyde in sodium cacodylate buffer (0.1 M; pH 7.4). After dehydration in graded ethanol and propylene oxide, the tissue blocks were embedded in Epon. Semithin sections were cut for light microscopy and stained with 0.1% (w/v) toluidine blue in 2.5% (w/v) sodium carbonate. These sections again were classified by a pathologist. Ultrathin sections were cut from defined areas of the tissues, stained with uranyl acetate and lead citrate and examined in a Zeiss EM910 (Carl Zeiss, Oberkochen, Germany) electron microscope. Electron micrographs were taken at 8,000x or 12,500x magnifications and scanned for digital processing with a Flextight X5 scanner (Hasselblad, Gothenburg, Sweden).

Of each patient, three blocks of each category available were examined. Per block two representative micrographs of non-overlapping regions were chosen and analyzed for the presence of an intact luminal glycocalyx, i.e. the height of the glycocalyx layer covering the epithelial cells was measured (5 values/micrograph) and the length of enterocyte surface with and without a glycocalyx was determined using image analysis and processing software (ImageJ (V.1.45b), NIH, Bethesda, MD; CorelDraw (V.11.0), Corel Corporation, Ottawa, Canada).

### Microparticle binding to caco-2 cells

Cholera toxin B subunit- (CTB) or *Eschericia coli* enterotoxin B subunit (LTB)-coated microparticles were prepared by coupling biotinylated CTB or LTB to avidin-coated, carboxylated fluorescent polystyrene particles (1 μm nominal size; Molecular Probes, Life Technologies, Darmstadt, Germany) as described previously [7].

Caco-2_BBe2_ cells (ATCC, Manassas, VA) were seeded on glass coverslips and cultured in Dulbecco’s modified Eagle medium (DMEM) containing 4 mM glutamine, 10 μg/ml transferrin, 10% (v/v) fetal calf serum (Hyclone, GE Healthcare Life Sciences, South Logan, UT) at 37°C under 10% CO_2_. After 20–26 days (14–20 days post confluence), cells were washed 4 x with phosphate-buffered saline and incubated with 5 x 10^7^/ml CTB- or LTB-coated microparticles in DMEM/glutamine/transferrin for 2 h at 37°C under 10% CO_2_. Cells were again washed with phosphate-buffered saline, fixed with 3% (w/v) formaldehyde, blocked with 0.2% (w/v) gelatin in phosphate-buffered saline, counterstained with 20 μg/ml fluorescein-labeled lectin (*Erythrina cristagalli* agglutinin (ECA), *Ulex europaeus* I agglutinin (UEA I), or *Ricinus communis* I agglutinin (RCA I), all from Vector Laboratories, Burlingame, CA) for 3 h at room temperature, washed 3 x with phosphate-buffered saline and mounted on glass slides. For examination of the differentiation state, formaldehyde-fixed cells were incubated with 20 μg/ml biotinylated lectin (ECA, UEA I, RCA I, as well as lectins from *Canavalia ensiformis* (Con A), *Maackia amurensis* (MAA), *Vicia villosa* (VVA), *Triticium vulgaris* (WGA), *Arachis hypogaea* (PNA), *Lens culinaris* (LCA), *Euonymus europaeus* (EEA)), and lectin binding was visualized by incubation with 5 μg/ml tetramethylrhodamine-labeled streptavidin.

### Statistical analysis

The electron micrographs were inspected independently by two persons. Statistical analyses were performed using Prism (V.5.01, GraphPad Software, Inc., La Jolla, CA). Normal distribution was checked with D’Agostino & Pearson omnibus and Kolmogorov-Smirnov tests. Differences between two groups were tested with Mann-Whitney test. Receiver-operator characteristics (ROC) curves were generated and the areas under the ROC curves (AUC) were determined to judge the adequacy of the apical glycocalyx as a marker to differentiate healthy tissue from early pathological changes (adenomas) and cancerous tissue (adenocarcinomas). The sensitivity and the specificity of the marker were determined at the optimal cut-point of each ROC curve. A probability of p < 0.05 was considered significant.

## Results

### Glycocalyx height on healthy tissue

We analyzed samples of 16 adenocarcinomas and 3 adenomatous polyps for the presence and thickness of an apical glycocalyx. Tissues that had been classified via pathological inspection as healthy mucosa served as controls (n = 15). The height of the existent glycocalyx-layer of all healthy specimens was measured in order to identify a cut-off value for discrimination between “intact” and “reduced” or “absent” glycocalyx (Table 1). As we found that the glycocalyx of the distal colorectum was significantly higher than of the proximal colon (Mann-Whitney test, p < 0.001), we divided the colorectum into two subsites. The right-sided colon included the cecum, ascending colon, right flexure, and transverse colon and had a mean glycocalyx height of 908 nm (n_random inspection_ = 130). The left flexure, descending colon, sigmoid colon and rectum belonged to the left-sided colorectum with a mean glycocalyx height of 1,254 nm (n_random inspection_ = 300). For both subsites the glycocalyx height followed a Gaussian distribution (D’Agostino & Pearson omnibus and Kolmogorov-Smirnov normality tests, p > 0.10 in right-sided colon and p > 0.05 in left-sided colorectum).

### Coverage of healthy and neoplastic tissue with glycocalyx

**Fig 1:**
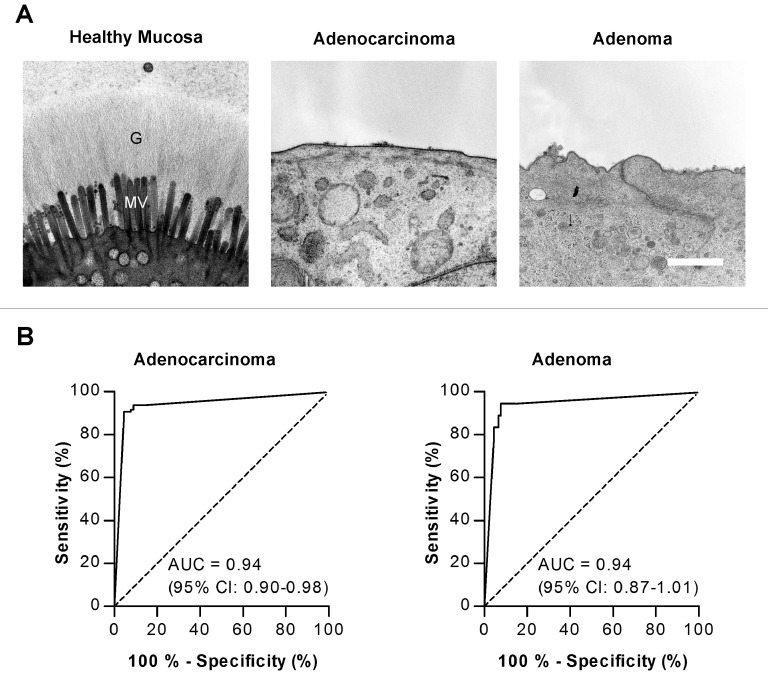
Glycocalyx of healthy as well as neoplastic epithelium in human colon and ROC analysis of the absence of a glycocalyx as a marker for colorectal cancer. (A) Transmission electron micrographs of enterocytes from healthy mucosa, adenocarcinoma and adenoma. The microvilli (MV) of healthy cells are covered by a thick glycocalyx (G), whereas the glycocalyx and microvilli are missing on neoplastic cells of adenocarcinomas and adenomatous polyps. Scale bar = 1 μm. (B) ROC curves for the absence of a glycocalyx as a marker in adenocarcinomas and adenomas. To judge the discriminatory power of this marker the AUC was calculated.

We chose the 1% percentiles (right-sided colon 426 nm, left-sided colorectum 616 nm) of the healthy glycocalyx height distributions to serve as cut-off values in order to classify the apical surface of the cells into sections that a) showed an intact glycocalyx (>1% percentile) or b) showed no intact glycocalyx (<1% percentile), i.e. to determine the coverage of the cells with an intact glycocalyx (Table 1). Overall, 93% (88–98%) (mean and 95% confidence interval) of the apical side of enterocytes located in healthy tissue were covered by an intact, thick glycocalyx, whereas this was the case for only 9% (4–15%) and 12.5% (-2-27%) of the apical surface of enterocytes in adenocarcinomas and adenomas, respectively. Hence, although some glycocalyx was visible on neoplastic cells, >90% (mean of all neoplastic specimens) of their apical surface was glycocalyx-free. The coverage of enterocytes with intact glycocalyx was significantly different between healthy and pathologic specimens (Mann-Whitney test, p < 0.001). The striking disparity in the surface architecture of healthy and degenerated colon tissue is illustrated in Fig 1A. The enterocytes of healthy colon tissues displayed regular, closely packed microvilli, and the glycocalyx mostly appeared as a thick, continuous surface coat covering the tips of the microvilli. In contrast, the enterocytes of cancerous tissues had few, in most cases no microvilli, and an intact glycocalyx was almost always absent. Even on adenomatous polyps, which can be considered a precursory stage of CRC, a distinctive glycocalyx was already missing.

To determine the sensitivity and specificity of the biomarker “absence of a glycocalyx” we analyzed the coverage of the apical side of the tissue of healthy and neoplastic cells by ROC analysis (Fig 1B). For adenocarcinomas and adenomas the AUC were 0.94±0.04 and 0.94±0.07, respectively, emphasizing the excellent performance of our marker. At the optimal cut-point of the ROC curve to least misclassify healthy and cancerous tissues for adenocarcinomas (<40.0% coverage with intact glycocalyx) sensitivity was 91% (83–96%) and specificity was 96% (89–99%). At the optimal cut-point of the ROC curve for adenomas (<62% coverage with intact glycocalyx) sensitivity was 94% (73–100%) and specificity 92% (85–97%) (means and 95% confidence intervals).

### Binding of particulate probes to membrane receptors covered by a glycocalyx

**Fig 2:**
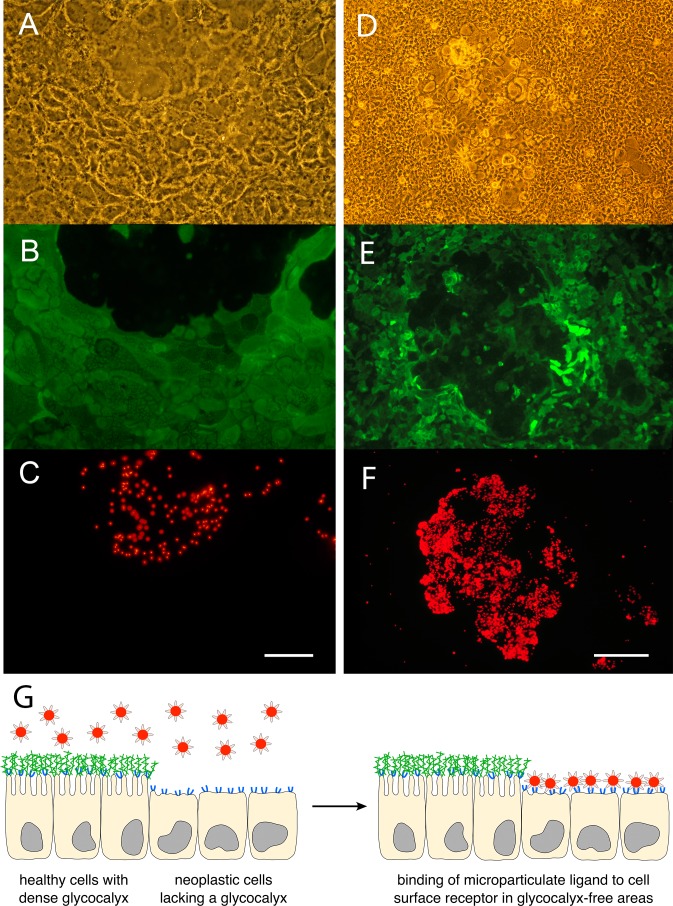
Detection of non-differentiated, glycocalyx-free enterocytes by particulate contrast agents. Exposure of the apical surface of variably differentiated areas of the human colon carcinoma cell line Caco-2_BBe2_ with CTB-coated (A-C) or LTB-coated (D-F) microparticles. (A, D) Phase contrast. (B, E) Visualization of the carbohydrate coat on partially differentiated cells with the fluorescein-labeled (green) lectin ECA (B) or UEA I (E). (C, F) Binding of CTB- (C) or LTB- (F) coated microparticles (red) to the membrane receptor ganglioside G_M1_ in glycocalyx-free areas of non-differentiated cells. Scale bar = 50 μm (A-C) respectively 200 μm (D-F). (G) Schematic illustration of the aspired *in vivo* detection of mucosal neoplasia. To detect CRC a particulate contrast agent (red) coated with a ligand for a cell membrane receptor (blue) can be used. In the intestine, the particles should bind selectively to the membrane receptors of anomalously differentiated cells that lack a glycocalyx (green). The particle-stained neoplasia can be visualized by appropriate imaging modalities.

To evaluate if the absence of a glycocalyx on tumor cells may be exploitable as a diagnostic target, e.g. by utilizing appropriate contrast agents in combination with imaging modalities, we tested this approach in a cell culture system. The human colon carcinoma cell line Caco-2_BBe2_ was used as *in vitro* cancer model. We established culture conditions under which the Caco-2_BBe2_ cells displayed a non-uniform differentiation pattern, forming monolayers of partially differentiated epithelial cells with short microvilli and a rudimentary glycocalyx, scattered with small islets of rather flat, non-differentiated cells without glycocalyx. In preliminary experiments, the presence/absence of the carbohydrate coat (glycocalyx) on the cells was verified by staining with 10 different lectins with binding specificities for different carbohydrate structures. In accordance with published data [2], all lectins used bound to 80 - >95% of the partially differentiated monolayer cells, indicating the presence of core-glycosylation as well as various terminal oligosaccharides. Areas of flat, non-differentiated Caco-2_BBe2_ cells exhibited no or only very sparse lectin binding, substantiating the essential lack of a glycocalyx in these regions. As model contrast agents we used fluorescent microparticles (nominal diameter: 1 μm) which were surface-modified with the B subunit of either cholera toxin (CTB) or *Escherichia coli* enterotoxin (LTB). Both toxin subunits are known ligands of ganglioside G_M1_, a receptor which is located in the epithelial cell membrane, i.e. underneath the glycocalyx. With each of the two microparticle-types, five independent binding-experiments with cultured Caco-2_BBe2_ cells were performed; the actual differentiation state of the cell areas was visualized by counterstaining with fluorescently labeled lectins UEA I, RCA I or ECA. Exposure of the variably differentiated Caco-2_BBe2_ cells to the CTB- or LTB-coated microparticles resulted in a distinct patchy binding pattern. In all cell layers investigated (n = 10), specific, clustered particle binding to cells was observed solely in those areas which were not stained by the lectins and hence lacked a glycocalyx (Fig 2). The carbohydrate coat which was present on partially differentiated cells could obviously not be penetrated by 1 μm particles and therefore efficiently prevented binding of the immobilized CTB or LTB to ganglioside G_M1_ in the epithelial cell membrane.

## Discussion

CRC is the third most diagnosed cancer and ranks third in cancer induced deaths in the United States [8]. Periodic screening can reduce the mortality of CRC, because the disease is well curable if recognized and treated early [9, 10], as is reflected in the 5-year survival rate for patients diagnosed with CRC, which is 93% for stage I CRC (early, localized stage) [11]. Yet, only 39% of CRCs are detected at an early, localized stage [12]. This is due to the low sensitivity of current diagnostical tests such as fecal occult blood tests (FOBT), and to the reluctance of many people to undergo more sensitive invasive tests such as flexible sigmoidoscopy or colonoscopy. Therefore it is highly desirable to develop alternative, ideally noninvasive diagnostical means for the early detection of CRC.

Our aim was to investigate whether the absence of a glycocalyx on the apical surface of neoplastic cells can serve as a biomarker for the detection of CRC and precancerous lesions, i.e. whether discrimination between healthy and neoplastic cells of the intestinal mucosa by this criterion is possible. Indeed, we could verify an excellent diagnostic potential of this nonclassical marker. Identification of neoplastic cells was possible for adenocarcinomas and adenomatous polyps with a sensitivity of 91% and 94% and a specificity of 96% and 92%. If this marker can be combined with an appropriate sensitive and specific noninvasive detection method, this could provide a highly promising alternative to current screening tests for CRC such as the three most commonly used tests FOBT, sigmoidoscopy and colonoscopy, which have sensitivities of 16–87%, 70–99% and 92–100%, respectively, and specificities of 86–98%, 60–92% and 43–90% for advanced colonic neoplasia (adenomas >10 mm in diameter, villous adenomas, adenomas with high-grade dysplasia, or invasive cancer) [13–15].

Besides CRC other diseases of the intestine exist where the epithelial glycocalyx is decreased, e.g. in the inflammatory diseases ulcerative colitis and celiac disease [16, 17]. Even in the healthy epithelium of the colon and rectum M cells are present [18], which per se have a loose glycocalyx network [2], and in some of the analyzed healthy tissues we found that the epithelium was not continuously covered by an intact glycocalyx. Thus, the absence of a glycocalyx does not necessarily indicate neoplasia. Such situations could diminish the specificity of our marker, as the absence of a glycocalyx on non-neoplastic cells might yield false positive results in a CRC screening where particulate contrast agents bind to epithelial cells lacking a glycocalyx. Yet, patients with highly inflammatory conditions of the intestines would in any case undergo regular (endoscopic) examinations to counteract their increased risk of intestinal carcinomas and hence be not the typical target group for a CRC screening test. Supportive of the proposed diagnostic system might also act the natural epithelial turnover–the healthy epithelium is renewed every 3 to 5 days via cell shedding, and in ulcerative colitis the turnover is even faster [19, 20]—which would lead to excretion of any particulate matter attached to healthy or inflamed epithelium within a short time span. In contrast, neoplastic epithelial cells are less subject to apoptosis and shedding [21, 22]. Therefore the anomalously differentiated, “immortal” cancer cells should retain any bound particles *in loco* for longer time periods. A specific diagnosis might hence be possible, simply by choosing the appropriate time frame between application of contrast agent and image capture in order to gain high sensitivity and specificity.

As yet, we can only speculate about the *in vivo* or even clinical potential of our marker “absence of a glycocalyx”. However, we have strong evidence that cell-surface receptors (e.g. gangliosides), which are not shielded by a glycocalyx in neoplastic cells, may be utilized in this regard. Our *in vitro* experiments with Caco-2_BBe2_ cells clearly demonstrate that microparticulate probes directed against an apical cell membrane receptor are prevented from reaching their target if a glycocalyx is present and only accumulate on glycocalyx-free areas of non-differentiated cells. This shall pave the way for the development of tumor-specific contrast agents that are assembled from a membrane receptor-targeting ligand and a particulate imaging reporter. Ideally, such a construct should allow noninvasive *in vivo* imaging of CRC. A very elegant method would be the use of superparamagnetic iron oxide nanoparticles that can be visualized by magnetic particle imaging (MPI) [23]. MPI is a new imaging modality that promises high sensitivity, resolution and imaging speed [24]. MPI enables a resolution of <1 mm [25], is noninvasive and does not involve exposure to high energy radiation or radioactive contrast agents, which are detrimental to patient compliance in screening programs. Our envisioned contrast agents could be administered orally and should pass the intestine without any adverse effects. Particle binding would occur only to the apical surface of neoplastic epithelium—if present—in the colon. Surplus, non-bound particles should be excreted without entering the body, thus minimizing any non-specific background in the subsequent imaging process.

In the resected tissues of the fifteen patients included in this study, only three adenomas were found that could be analyzed for the presence and height of a glycocalyx. In spite of this small number we decided to include these adenomas in the study to get some indications regarding the potential usability of our marker for the detection of precursory stages of CRC. We would like to point out that the number of analyzed adenomas is too small to get statistically reliable results, therefore the drawed conclusions should be seen with caution. Yet, in the small number of adenoma samples analyzed, we also achieved excellent sensitivity and specificity values for the marker “absence of a glycocalyx” which suggests that morphological changes in the glycocalyx layer might occur early in CRC development. The absence of a glycocalyx already in these precancerous lesions indicates that our marker might be suitable for CRC screening. Yet, when considering the data, one has to bear in mind that there are diverse types of adenomas developing via different pathways. They can be classified as conventional (tubular, tubulovillous, villous) and serrated (sessile serrated adenomas, traditional serrated adenomas, hyperplastic polyps) adenomas [26, 27]. The three adenomas analyzed in this study belonged to the conventional types. In future investigations regarding the *in vivo* suitability of the marker “absence of a glycocalyx” for the early detection of CRC special attention would have to be given to the diverse types of adenomas.

In conclusion, we have identified the absence of an intact glycocalyx on neoplastic cells as a novel and auspicious biomarker for early detection of CRC, and demonstrated its suitability for the localization of glycocalyx-free intestinal epithelial cells in an *in vitro* cell culture model.
